# Evaluating genetic-ancestry inference from single-cell RNA-seq data

**DOI:** 10.1101/2025.03.25.645175

**Published:** 2025-03-28

**Authors:** Jianing Yao, Steven Gazal

**Affiliations:** 1 Department of Population and Public Health Sciences, Keck School of Medicine, University of Southern California, Los Angeles, CA, USA; 2 Center for Genetic Epidemiology, Keck School of Medicine, University of Southern California, Los Angeles, CA, USA; 3 Department of Biostatistics, Bloomberg School of Public Health, Johns Hopkins University, Baltimore, MD, USA; 4 Department of Quantitative and Computational Biology, University of Southern California, Los Angeles, CA, USA

## Abstract

Characterizing the ancestry of donors in single-cell RNA sequencing (scRNA-seq) studies is critical to ensure the genetic homogeneity of the dataset and reduce biases in analyses, to identify ancestry-specific regulatory mechanisms and understand their downstream role in diseases, and to ensure that existing datasets are representative of human genetic diversity. While scRNA-seq is now widely available, the information on the ancestry of the donors is often missing, hindering further analysis. Here we propose a framework to evaluate methods for inferring genetic-ancestry from genetic polymorphisms detected from scRNA-seq reads. We demonstrate that widely used tools (e.g., ADMIXTURE) provide accurate inference of genetic-ancestry and admixture proportions despite the limited number of genetic polymorphisms identified and imperfect variant calling from scRNA-seq reads. We inferred genetic-ancestry for 196 donors from four scRNA-seq datasets from the Human Cell Atlas and highlighted an extremely large proportion of donors of European ancestry. For researchers generating single-cell datasets, we recommend reporting genetic-ancestry inference for all donors and generating datasets that represent diverse ancestries.

## Introduction

Gene expression can vary across individuals from diverse human ancestries due to heterogeneous genetic and environmental backgrounds ^1–10^, potentially contributing to disparities in disease mechanisms and susceptibility ^10^. Therefore, characterizing the ancestry of donors in transcriptomic studies is critical not only to ensure genetic homogeneity of the dataset and reduce biases in analyses, but also to identify ancestry-specific regulatory mechanisms and understand their downstream role in diseases.

Single-cell RNA sequencing (scRNA-seq) has emerged as a powerful technique to estimate gene expression within individual cells and has enabled the characterization of transcriptome profiles of multiple human cell types ^11^. Hundreds of scRNA-seq datasets across diverse tissues and conditions are now publicly available, for example through the Human Cell Atlas ^11^ (HCA) portal ^12^, but the lack of donor ancestry information impedes further investigative work. Detecting genetic polymorphisms in scRNA-seq reads offers a unique opportunity to characterize genetic-ancestry (defined as the biological descendance from various ancestral groups) of donors without access to additional genetic data ^13,14^. However, the accuracy of this strategy remains unknown due to the limited fraction of the genome that is transcribed and the noise in scRNA-seq data that impacts variant calling accuracy ^13^.

Here we propose a framework to evaluate methods for inferring genetic-ancestry from scRNA-seq data using individuals from both the Human Genome Diversity Project ^15^ (HGDP) and the 1000 Genomes Project ^16^ (1kGP) as a reference dataset ^17^. We demonstrate that widely used tools (e.g., ADMIXTURE ^18^) provide accurate inference of genetic-ancestry and admixture proportions despite the limited number of variants and imperfect variant calling from scRNA-seq reads. We inferred genetic-ancestry for 196 donors from four scRNA-seq datasets ^19–22^ from the HCA and highlighted an extremely large proportion of donors of European ancestry. We recommend that researchers generating single-cell datasets document the genetic-ancestry of the donors and focus on including donors from diverse ancestries.

## Material and methods

### Common SNP calling in scRNA-seq data

We identified genetic variants from scRNA-seq data using GATK ^23,24^ as recommended in ref. ^25^; this pipeline also provided the highest genotyping accuracy in single-nucleus RNA-seq data compared to other methods in ref. ^13^. We used the RNAseq short variant discovery workflow as the following. First, the processed sequencing reads were aligned to the human hg19 reference genome using STARSolo v2.7.11a in a two-pass mode to achieve better alignments around novel splice junctions (SJs). In the first pass, SJs were identified across all samples by combining unique and multi-mapped reads, and then filtered to exclude SJs supported only by multi-mapped reads or with a unique count below two. The filtered SJ file was then used in the second pass, with the STAR output coordinate-sorted. Second, we combined the BAM files of all cells from each individual to create a pseudo-bulk BAM file for each donor. Duplicate reads were flagged using GATK’s MarkDuplicates, and GATK’s SplitNCigarReads was applied to reformat alignments spanning introns, ensuring compatibility with HaplotypeCaller for variant calling. Third, variants of each donor were called using GATK’s HaplotypeCaller. Finally, joint genotyping of all donors from each HCA dataset was performed using GATK’s GenotypeGVCFs, followed by filtering with GATK’s VariantFiltration, which removed sites with low QualByDepth <2, high FisherStrand > 60, StrandOddsRatio > 3, RMSMappingQuality < 40, MappingQualityRankSumTest < −12.5, and ReadPosRankSumTest < −8.

We performed additional quality control (QC) steps to optimally select SNPs and samples. To retain only SNPs that are informative for genetic-ancestry inference while limiting false-positive genotypes, we kept those SNPs that were common (minor allele frequency (MAF) > 5%) in the QCed HGDP+1kGP dataset (see below) and located within coding exons and untranslated regions (UTRs) (defined using UCSC reference files and extended by 150 base pairs upstream and downstream). We used PLINK to exclude SNPs with genotype missingness higher than 10% and donors with genotype missingness higher than 10%.

We have released an open-source pipeline implementing our framework (see Code availability).

### scRNA-seq datasets

We inferred genetic-ancestry for 196 donors from four HCA scRNA-seq datasets (Table 1). At the beginning of the study, these datasets represented the HCA datasets with the highest number of donors with unreported ancestry. These datasets encompassed diverse tissues and cell types and employed different sequencing technologies (i.e., Smart-seq, Drop-seq, and 10X Genomics 3’ sequencing), enabling a comprehensive evaluation of genetic-ancestry inference across varying combinations of genes expressed and technical platforms. To evaluate the genotype error rate of our pipeline, we leveraged 12 individuals with both scRNA-seq and SNP-array data from ref. ^26^; where we considered the SNP-array genotypes as ground truth.

### Genetic-ancestry inference approaches

We considered the harmonized HGDP+1kGP dataset ^17^ as a reference population dataset for genetic-ancestry inference and selected six genetic-ancestry groups (Africa, America, Europe, Middle East, East Asia, and South Asia; note that the Finnish population was included within the European group). Several QC steps were applied before downstream analysis. Specifically, we removed individuals who were outliers in principal component analyses of their reported genetic-ancestry groups to obtain homogeneous reference populations. The final dataset consisted of 3,481 individuals from 67 populations (Supp. Table 1). Further analyses were restricted to SNPs common (MAF > 5%) in this dataset.

We considered three commonly used approaches to infer genetic-ancestry of a studied sample (i.e., donors from a scRNA-seq study) using a reference dataset (i.e., HGDP+1kGP). Two approaches relied on a principal component analysis (PCA) performed on HGDP+1kGP with the projection of donors on the five first principal components (PCs). First, we assigned each donor to the genetic-ancestry group with the smallest Euclidean distance to its centroid (method labeled PCA-Distance). Second, we used a random forest classifier trained on the five PCs to predict genetic-ancestry probabilities for each study sample, as performed in ref. ^17^ (method labeled PCA-RandomForest). Finally, we applied ADMIXTURE ^18^ in supervised mode using the HGDP+1kGP genetic-ancestry groups as reference (e.g., K=6) to estimate the proportions of ancestral populations for each donor. These three approaches were run after linkage disequilibrium (LD)-pruning performed with PLINK ^27^ (--indep-pairwise option with a window size of 50 SNPs, a step size of 10 SNPs, and an *r*^2^ threshold of 0.1) on the HGDP-1kGP dataset restricted to the investigated SNPs (between 2,968 and 7,614 SNPs after LD pruning; see Table 1).

### Evaluating genetic-ancestry inference using HGDP+1kGP

We evaluated the three genetic-ancestry inference approaches using a leave-one-population-out strategy on HGDP+1kGP. Specifically, for each of the 67 HGDP+1kGP populations, we removed the corresponding individuals from the reference dataset and inferred genetic-ancestry of these people using each approach to evaluate the robustness of the inference methods when prior information about the excluded population was unavailable. We repeated this procedure for every population. For PCA-RandomForest and ADMIXTURE, we assigned each individual from the removed population to the genetic-ancestry category corresponding to the highest predicted proportion. Finally, we compared the inferred genetic-ancestry to that provided by the HGDP+1kGP dataset and evaluated the inference error rate both within each genetic-ancestry group and across all individuals.

We evaluated each approach using different sets of genotypes. First, to define a gold standard, we considered genotypes from all common SNPs in the HGDP+1kGP dataset, performed LD pruning, and retained 295,434 SNPs; we labeled this genotype dataset “all-SNPs”. Second, to evaluate genetic-ancestry inference from SNPs identified in scRNA-seq datasets, we considered four distinct sets of SNPs corresponding to the LD-pruned SNPs identified in the four HCA datasets; we labeled these genotype datasets “sc-SNPs”. Finally, to account for genotype detection error using scRNA-seq data, we leveraged the error rate estimated from the 12 individuals with both scRNA-seq and SNP-array data (8%; see Results) and simulated an 8% genotype error rate within each sc-SNPs dataset for individuals in the removed population; we labeled these genotype datasets “sc-SNPs-8%error”.

Finally, we evaluated the estimation of genetic-admixture proportions with ADMIXTURE using admixed individuals from the African Americans from the Southwest US (ASW) population of HGDP+1kGP. Specifically, we ran ADMIXTURE on the set of 295,434 LD-pruned SNPs (all-SNPs), selected 41 out of 68 ASW individuals with more than 1/8 European genetic-ancestry, and considered their proportion of European genetic-ancestry as the gold standard. Next, we reran ADMIXTURE on these individuals using genotypes from each set of sc-SNPs, as well as on genotypes where we simulated an 8% genotype error rate (sc-SNPs-8%error).

## Results

### Detecting common genetic polymorphisms in scRNA-seq datasets

We ran our pipeline on four scRNA-seq datasets from the HCA. After QC, we detected between 6,589 and 21,895 SNPs that were also present in the HGDP+1kGP dataset (average: 12,906), which were further reduced to between 2,968 and 7,614 common SNPs (average: 5,027) after genetic pruning (Table 1). We assessed the precision of SNP detection in scRNA-seq datasets by comparing the genotypes called in 12 individuals with both scRNA-seq data and genotype data (considered as ground truth). Across all individuals, we identified 13,900 genotypes from SNPs shared by both datasets, of which 12,819 were consistent, resulting in an overall error rate of approximately 8% (Supp. Table 2). We used this number to model the scRNA-seq genotype error rate in downstream analyses. We note that this rate is higher than that reported for GATK on single-nucleus RNA-seq ^13^ (~2%), likely due to improved RNA quality and reduced degradation that facilitate variant calling in these data.

### Quantifying genetic-ancestry inference from scRNA-seq data

**Figure 1.**
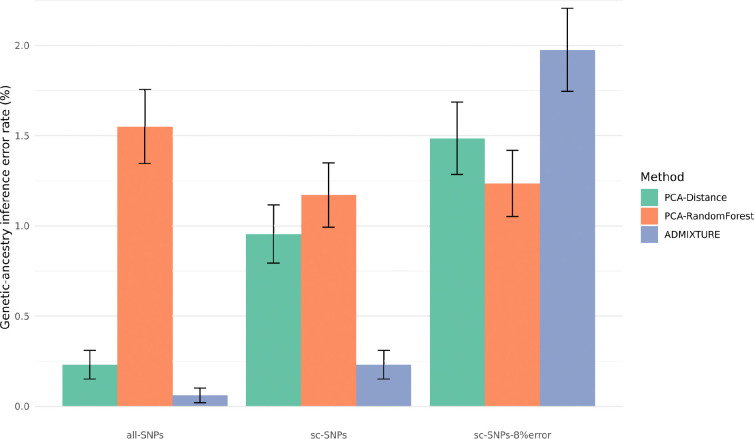
Genetic-ancestry inference error rate in HGDP+1kGP. We report genetic-ancestry inference error rates estimated using 3,481 HGDP+1kGP individuals from 67 populations spanning 6 ancestry groups. We considered three sets of genotypes for genetic-ancestry inference: genotypes from all common SNPs (all-SNPs; 295,434 common SNPs after pruning), genotypes from SNPs observed in scRNA-seq datasets (sc-SNPs), and genotypes from SNPs observed in scRNA-seq datasets for which we simulated an 8% genotype error rate (sc-SNPs-8%error). Genetic-ancestry inference error rates estimated using genotypes from sc-SNPs and sc-SNPs-8%error were averaged across four different sets of SNPs (see Table 1). Error rates estimated on genotypes from all-SNPs were considered the gold standard. Error rates for each genetic-ancestry group and for each set of SNPs are reported in Supp. Figure 1 and Supp. Figure 2, respectively. Error bars represent 95% confidence intervals. Numerical results are reported in Supp. Table 3.

We estimated genetic-ancestry inference error using a leave-one-population-out approach on the HGDP+1kGP dataset (Figure 1 and Supp. Table 3). Using the all-SNPs dataset as the gold standard, we observed that ADMIXTURE provided a negligible low error rate (0.06% across 3,481 HGDP+1kGP individuals), significantly lower than that obtained by PCA-Distance (0.23%) and PCA-RandomForest (1.55%). When restricting these analyses to SNPs identified in scRNA-seq datasets (sc-SNPs), ADMIXTURE still yielded an extremely low error rate (0.23% across the four datasets), at least about four times lower than those of PCA-Distance and PCA-RandomForest (0.91% and 1.17%, respectively). However, after simulating the genotype error rate in these datasets (sc-SNPs-8%error), the error rate for ADMIXTURE became higher than those for PCA-Distance and PCA-RandomForest, yet remained relatively low (1.98%, 1.49%, and 1.24%, respectively). Overall, the higher error rate for PCA-RandomForest was primarily driven by misclassification of individuals from the Druze and Mozabit populations, whereas the higher error rate for ADMIXTURE with sc-SNPs-8%error was mainly due to misclassification of individuals from the Tuscan population (in these scenarios, those individuals were inferred as an admixture of European and Middle Eastern genetic-ancestries; Supp. Figure 1). We observed similar trends across the four sc-SNPs datasets (Supp. Figure 2). Moreover, we found that the ADMIXTURE error rate increased exponentially with the genotype error rate (starting at error rates greater than 6%), whereas the error rate for PCA-based methods increased linearly (Supp. Figure 3). Finally, we investigated whether SNPs identified in scRNA-seq datasets allow for more fine-grained genetic-ancestry inference analyses. Specifically, we tested whether sub-population groups within an ancestry group could be inferred (e.g., identifying Japanese rather than simply East-Asian genetic-ancestry) ^28^. We observed that although ADMIXTURE can yield an acceptable error rate within some ancestry groups using sc-SNPs, all inference approaches produced high error rates when using sc-SNPs-8%error (Supp. Figure 4 and Supp. Figure 5). Overall, our results indicate that while inferring continental genetic-ancestry from SNPs in scRNA-seq data provides exceptionally low error rates with ADMIXTURE under ideal conditions, the error rate increases to relatively low values when accounting for a genotype error rate of 8%.

### Quantifying genetic-admixture inference from scRNA-seq data

**Figure 2.**
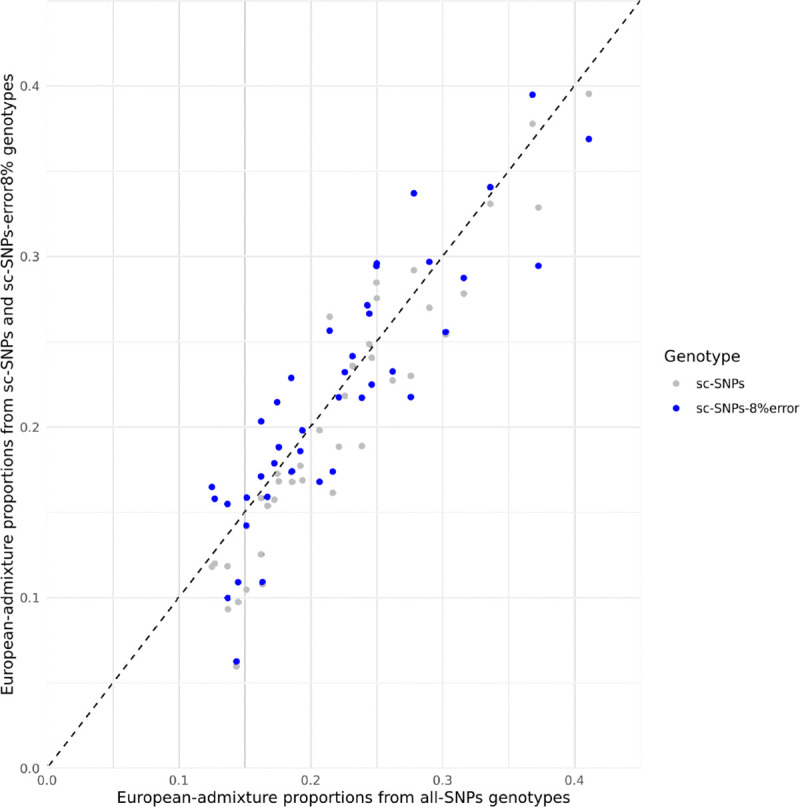
ADMIXTURE estimates of European-admixture proportions among ASW individuals. We report ADMIXTURE estimates of European-admixture proportions among 41 ASW admixed individuals using genotypes from all-SNPs (x-axis), sc-SNPs (y-axis, grey dots), and sc-SNPs-8%error (y-axis, blue dots). Proportions estimated using genotypes from sc-SNPs and sc-SNPs-8%error were averaged across four different sets of SNPs. Proportions estimated on genotypes from all-SNPs were considered the gold standard. The correlation between all-SNPs and sc-SNPs (resp. sc-SNPs-8%error) estimates is 0.94 (resp. 0.88), with a regression slope of 1.05 (resp. 0.91). Estimates using SNPs from each scRNA-seq dataset are reported in Supp. Figure 6. Numerical results are reported in Supp. Table 4.

Donors from scRNA-seq datasets are likely to be genetically admixed. We therefore evaluated genetic-admixture inference from ADMIXTURE by leveraging 41 admixed individuals from the ASW population, each with more than one-eighth of their genome estimated to be of European genetic-ancestry. We considered European genetic-admixture proportions estimated from all-SNPs as the gold standard and observed high consistency with those obtained using sc-SNPs (slope = 1.05, *r* = 0.94) and sc-SNPs-8%error (slope = 0.91, *r* = 0.88) (Figure 2 and Supp. Table 4). We reached similar conclusions across the four sc-SNPs datasets (Supp. Figure 6). Overall, these results suggest that ADMIXTURE can also provide robust estimates of genetic-admixture proportions.

### Estimating genetic-ancestry in 4 large datasets from the Human Cell Atlas

**Figure 3.**
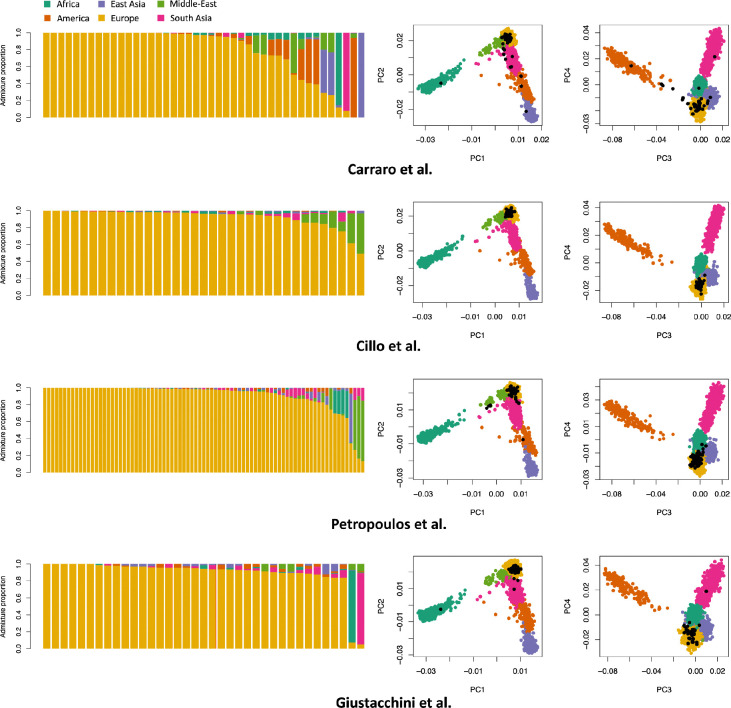
ADMIXTURE and PCA analyses of four HCA scRNA-seq datasets. The left panels show the admixture proportions of HCA donors from each dataset. The right panels display PCA plots of HGDP+1kGP individuals (colored dots) with the projection of HCA donors (black dots). Numerical results are reported in Supp. Table 5.

Finally, we used ADMIXTURE and PCA to estimate and visualize genetic-ancestry for 196 individuals (after QC) across four HCA datasets (Figure 3 and Supp. Table 5). Overall, we observed that these datasets were predominantly composed of donors with European genetic-ancestry: the mean European genetic-admixture proportion estimated by ADMIXTURE was 87.9% (median 96.5%), with 91.8% (resp. 84.2%) of the donors having a proportion >50% (resp. >80%). Across the four datasets, we identified only 2 individuals with >50% African genetic-admixture proportions, 3 with >50% American, 4 with >50% East Asian, 3 with >50% Middle East, and 2 with >50% South Asian. We observed nearly identical results with PCA-Distance (92.3% of individuals inferred as having European genetic-ancestry) and PCA-RandomForest (with 91.3% and 86.7% of individuals having a probability greater than 50% and 80%, respectively, of European genetic-ancestry) (Supp. Table 5). Overall, these results suggest limited representation of non-European populations in the publicly available scRNA-seq datasets.

## Discussion

Characterizing the ancestry of donors in scRNA-seq studies is essential to ensure genetic homogeneity of the dataset and to identify ancestry-specific regulatory mechanisms linked to disease risk. Here, we proposed a framework to evaluate methods for inferring genetic-ancestry from scRNA-seq data. We considered four realistic sets of SNPs that account for the variability in SNP detection across different single-cell technologies and human tissues, and we simulated genotype errors to reflect imperfect variant calling in scRNA-seq data. We demonstrated that ADMIXTURE provides accurate inference of genetic-ancestry and European admixture proportions in African-American individuals despite the limited number of genetic polymorphisms and imperfect variant calling from scRNA-seq reads. We anticipate that our easy-to-use pipeline (see Code availability) will enable researchers to infer and document the genetic-ancestry of their samples and facilitate the characterization of genetic-ancestry across publicly available scRNA-seq datasets. Finally, we observed an over-representation of donors with European genetic-ancestry in four HCA datasets, consistent with observations in RNA-seq datasets ^29^ and genome-wide association studies ^30^. Although recent studies have generated large scRNA-seq datasets using hundreds of Asian donors ^26,31^, there remains a need to generate scRNA-seq datasets in more genetically diverse populations to ensure that existing datasets are representative of human genetic diversity.

We note some limitations of our work. First, we considered six genetic-ancestry groups that are not fully representative of human genetic diversity. In addition, categorizing genetic-ancestry oversimplifies human genetic diversity and disregards our understanding of human demographic history ^32^. Second, we restricted our inferences to common SNPs identified in scRNA-seq reads by GATK, as calling variants at known loci decreases the probability of false positives, and GATK is a widely used tool that provides high genotyping accuracy compared to other methods ^13,25^. More sophisticated variant-calling methods ^13^ might improve the number of SNPs detected and/or genotype calling accuracy. However, even when simulating a conservative 8% genotype error rate on common variants, we observed that ADMIXTURE provides reliable results and can achieve extremely high genetic-ancestry inference accuracy for lower genotype error rates (Supplementary Figure 3). Finally, we analyzed only four datasets from HCA, which represented the datasets with the most donors of unreported ancestry at the time of the beginning of the study. Applying our pipeline to the complete HCA dataset (22,013 samples from 461 projects, as of 3/7/2025) would enable a comprehensive genetic-ancestry characterization of publicly available scRNA-seq data. Third, we investigated admixture estimation accuracy only by estimating European-admixture in an African-American population. Despite these limitations, our study provides a robust framework to evaluate methods for inferring genetic-ancestry from genetic polymorphisms detected from scRNA-seq reads.

## Supplementary Material

Supplement 1

Supplement 2

## Figures and Tables

**Table 1.** HCA scRNA-seq datasets analyzed in this study.

Dataset	Technology	Tissue	# donors before / after QC	# cells	# Qced SNPs before / after pruning
Carraro et al. ^19^	10X 3’ v2/v3, Drop-seq	lung epithelium, submucosal gland	50 / 43	Not reported	9,100 / 4,515
Cillo et al. ^20^	10X 3’ v2	CD 45+ cells from diverse tissues	37 / 35	126.0K	21,895 / 7,614
Petropoulos et al. ^21^	Smart-seq2	embryonic cell, inner cell mass cell	88 / 81	1,529	14,039 / 5,014
Giustacchini et al. ^22^	Smart-seq2	Bone marrow	42 / 37	2.3K	6,589 / 2,968

## Footnotes

Code availability Our pipeline and the code to replicate our analyses are available at https://github.com/JianingYao/scRNA-seq-genetic-ancestry.
