# Supplementary material for: Evaluating genetic-ancestry inference from single-cell RNA-seq data
Source: bioRxiv. 2025 Mar 28:2025.03.25.645175. Preprint. [Version 1] doi: 10.1101/2025.03.25.645175 (PMC11974901; doi:10.1101/2025.03.25.645175)
Supplement: Supplement 1 [file media-1.pdf]

Supplementary Table Legends

Supplementary Table 1. Description of the HGDP-1kGP reference dataset. We report the 3,481 HGDP-1kGP individuals used in our reference dataset, their genetic-ancestry groups (i.e., Africa, America, Europe, Middle East, East Asia, or South Asia), and their populations.

Supplementary Table 2. Estimation of genotype error rate in scRNA-seq data. We evaluated the genotype error rate of our pipeline by leveraging 12 individuals with both scRNA-seq and SNP-array data from ref. [26]. Genotypes from the SNP-array were considered the ground truth.

Supplementary Table 3. Genetic-ancestry inference error rate in HGDP+1kGP. We report the genetic-ancestry inference error rate and the corresponding 95% confidence interval (CI) estimated using 3,481 HGDP+1kGP individuals from 67 populations across 6 ancestry groups.

Supplementary Table 4. ADMIXTURE estimates of European-admixture proportions among ASW individuals. We report ADMIXTURE estimates of European-admixture proportions among 41 ASW admixed individuals using genotypes from all-SNPs, sc-SNPs, and sc-SNPs-8%error.

Supplementary Table 5. ADMIXTURE estimates on the four HCA scRNA-seq datasets. We report genetic-ancestry inference results obtained using three approaches, along with admixture proportions and random forest probabilities for HCA donors from each dataset.

Supplementary Figures

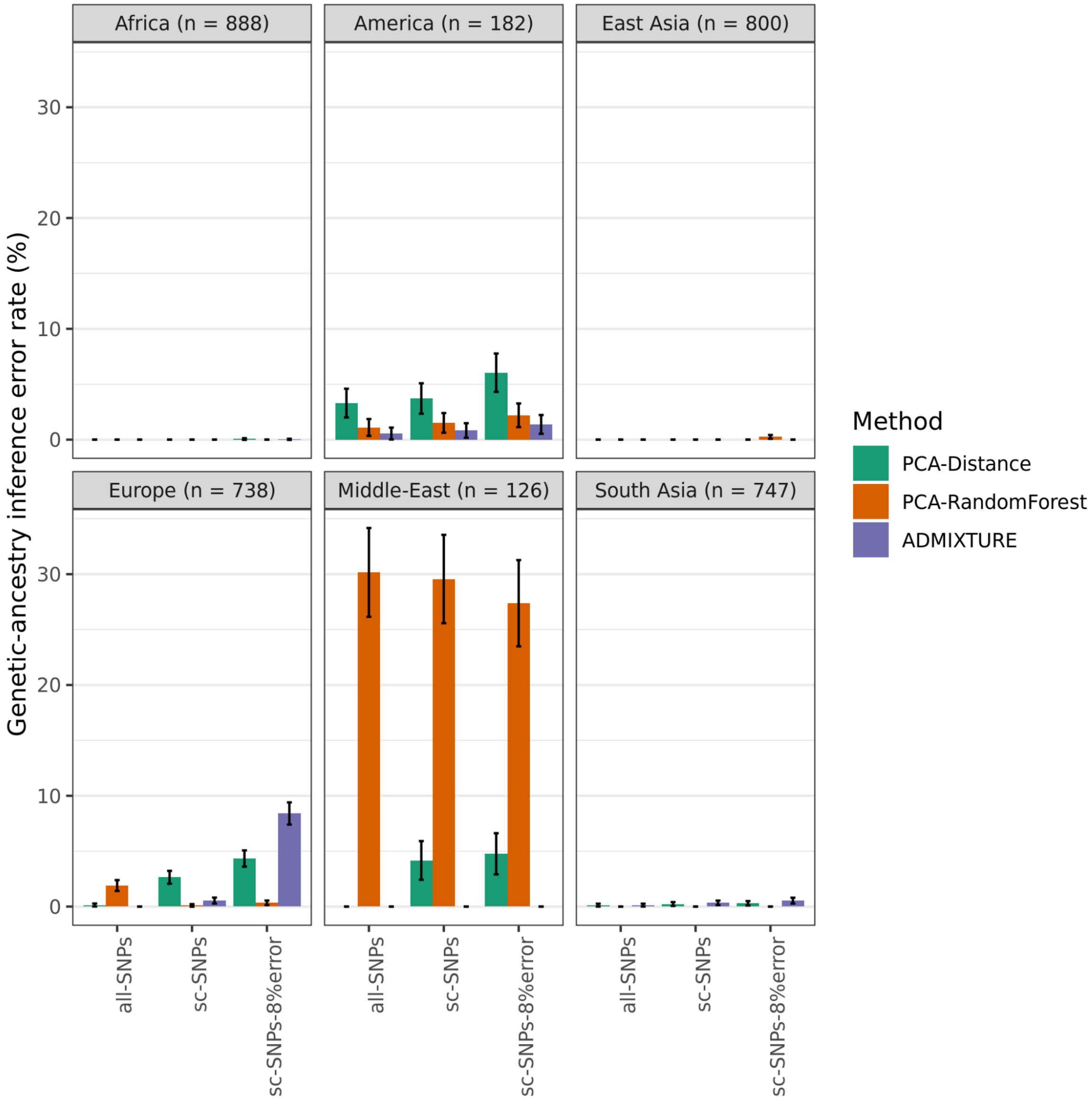

Supplementary Figure 1. Genetic-ancestry inference error rate stratified by ancestry group. We report the results from **Figure 1** stratified by ancestry group. Genetic-ancestry inference error rates estimated from genotypes using all-SNPs, sc-SNPs, and sc-SNPs-8%error were averaged across four different sets of SNPs. The higher error rate for PCA-RandomForest was primarily driven by misclassification of individuals from the Druze and Mozabit populations, and the higher error rate for ADMIXTURE with sc-SNPs-8%error was mainly due to misclassification of individuals from the Tuscan population (in these scenarios, those individuals were inferred as an admixture of European and Middle Eastern genetic-ancestries). Error bars represent 95% confidence intervals.

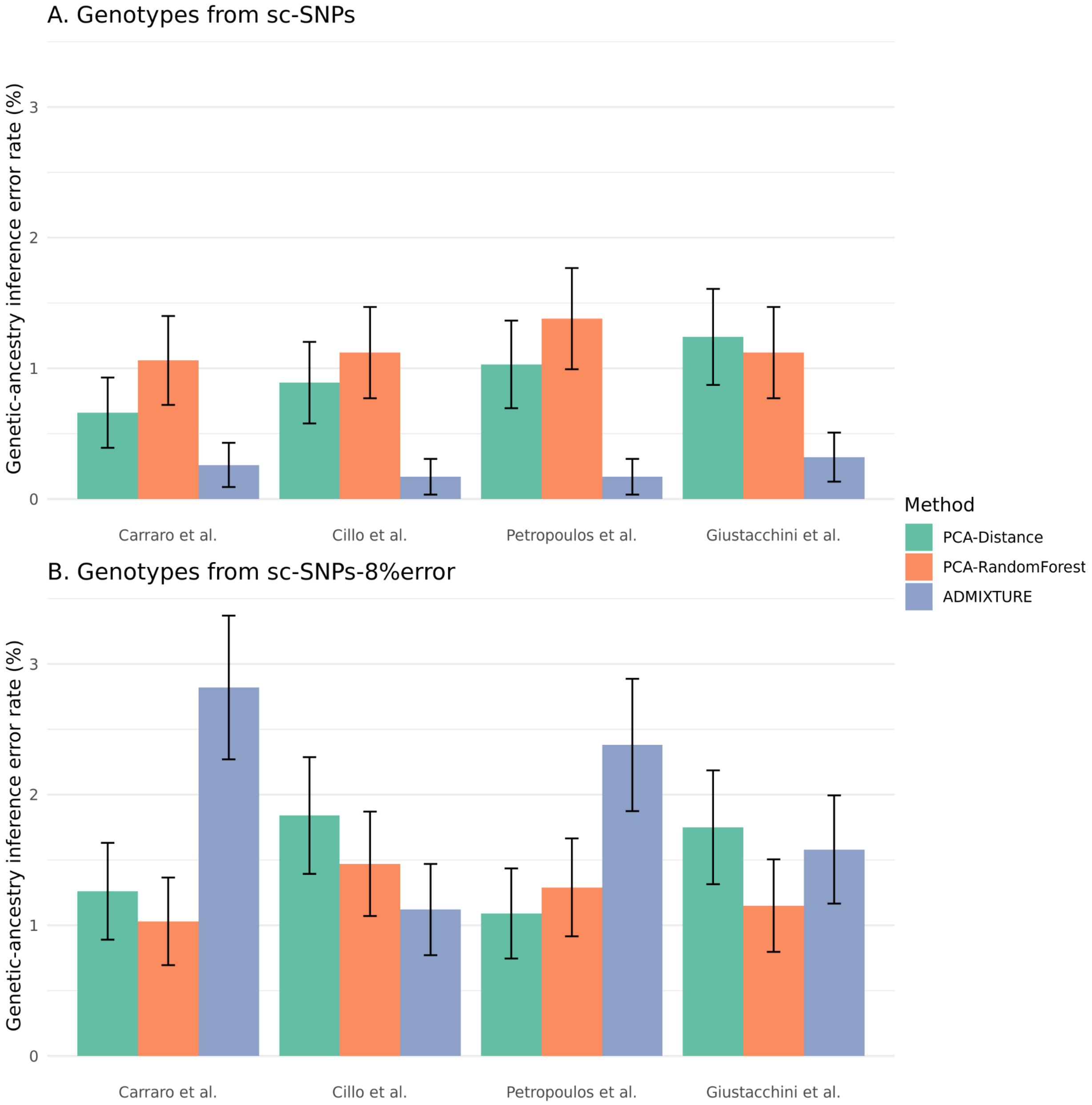

Supplementary Figure 2. Genetic-ancestry inference error rate stratified by scRNA-seq dataset. We report the results for genotypes from sc-SNPs (top panel) and sc-SNPs-8%error (bottom panel), stratified by scRNA-seq dataset. Error bars represent 95% confidence intervals.

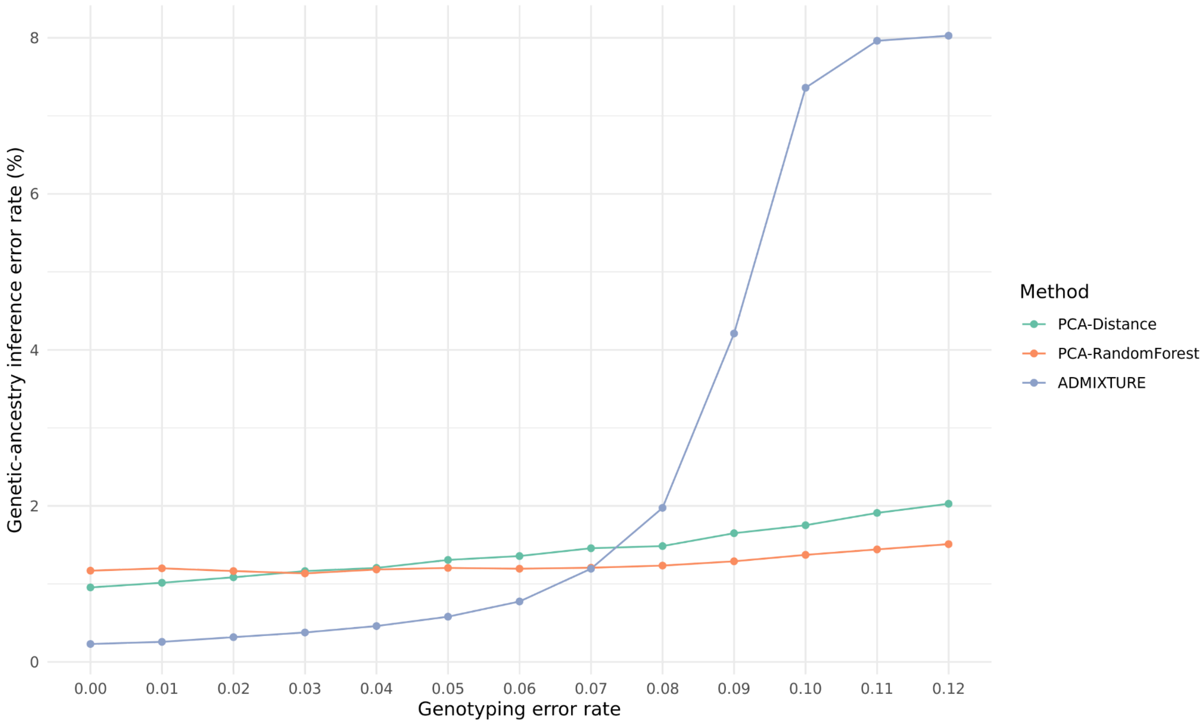

Supplementary Figure 3. Genetic-ancestry inference error rate as a function of genotyping error rate. For various genotyping error rates, we simulated errors within each sc-SNPs dataset for individuals in the removed population and reported the corresponding genetic-ancestry inference error rate.

A. Genotypes from all-SNPs

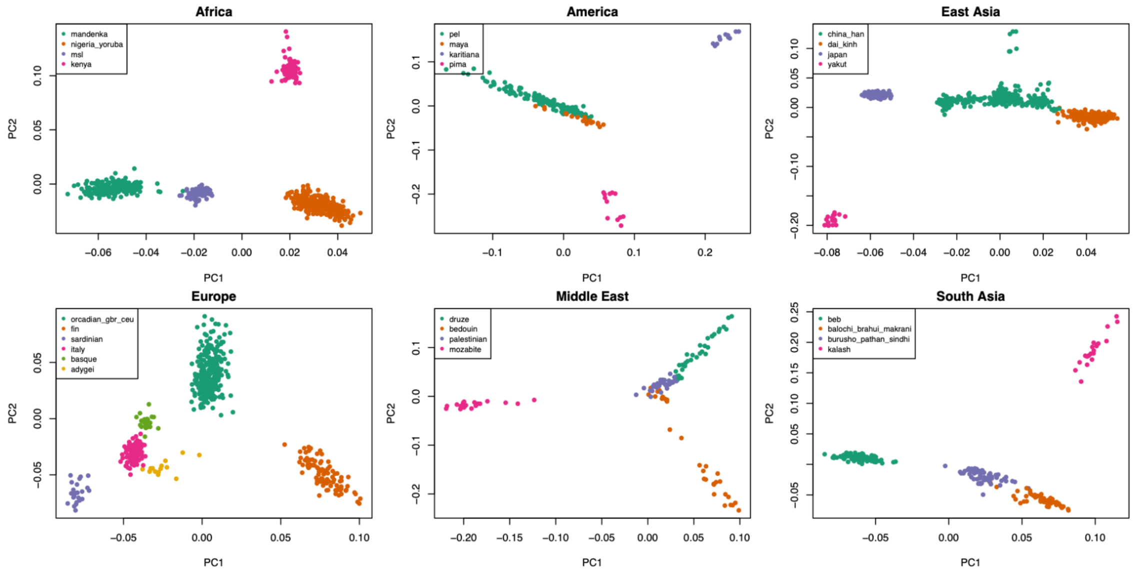

B. Genotypes from sc-SNPs from Giustacchini et al. dataset

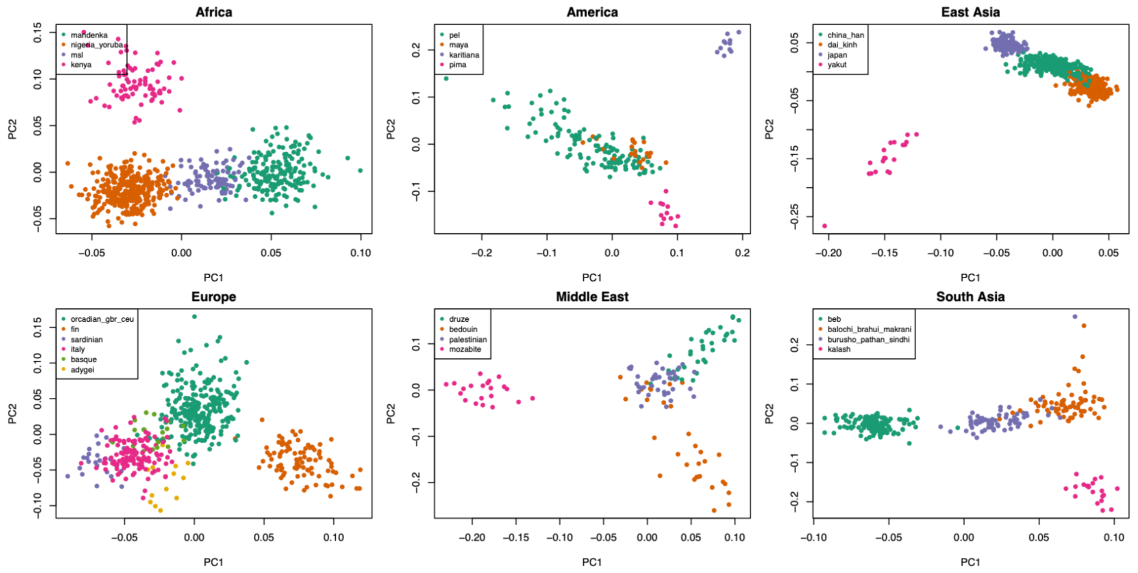

Supplementary Figure 4. Principal component analysis within each ancestry group. For each ancestry group, we performed a PCA using genotypes from (**A**) all-SNPs and (**B**) sc-SNPs from the Giustacchini et al. dataset. HGDP+1kGP populations were grouped into homogeneous populations. This figure illustrates the challenge of inferring sub-population groups within an ancestry group using sc-SNPs.

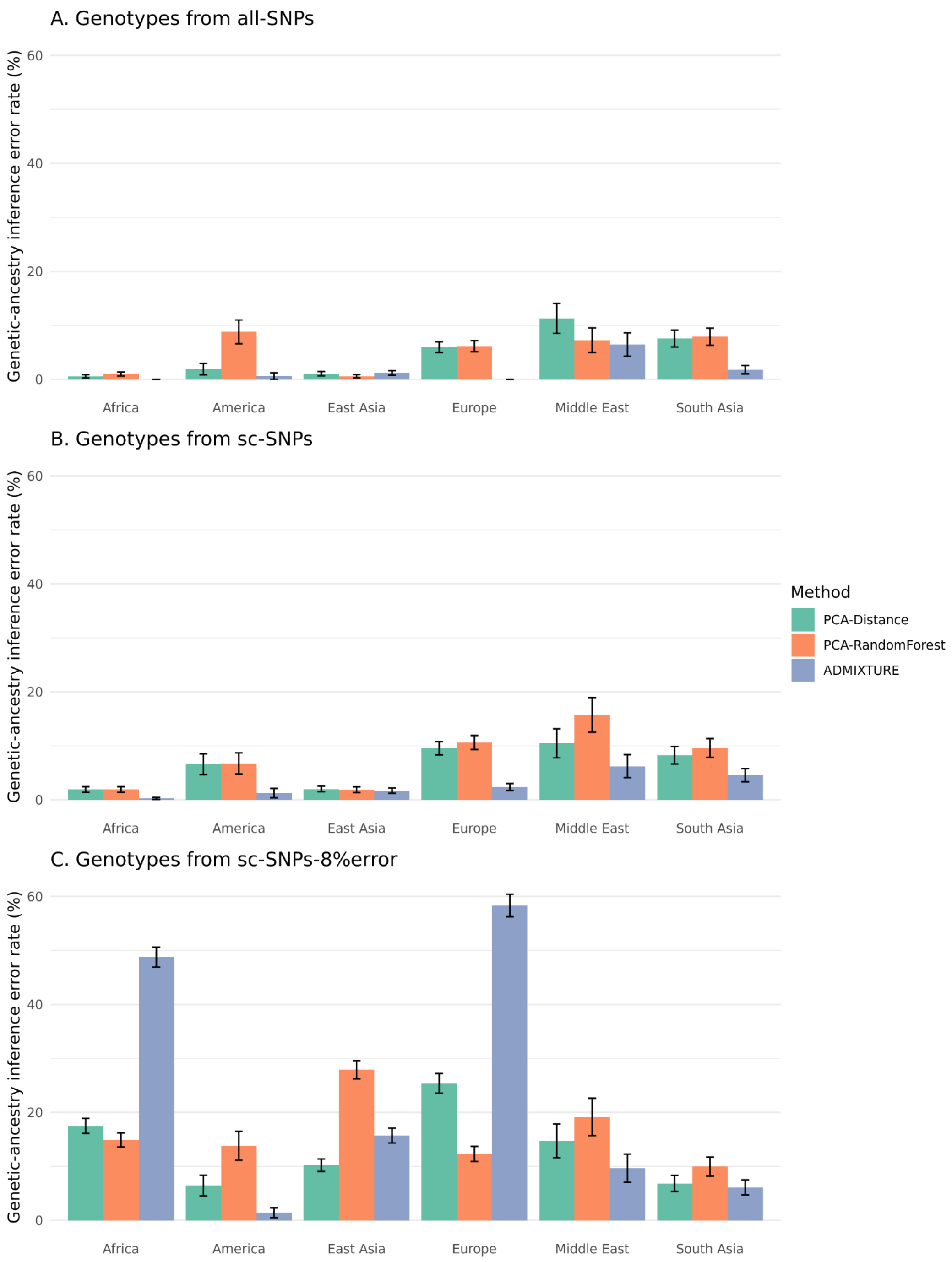

Supplementary Figure 5. Genetic-ancestry inference error rate within each ancestry group. For each ancestry group, we considered homogeneous populations (see **Supp. Figure 4**), divided the dataset into 10 splits while maintaining a similar number of individuals from each population in every split, and performed genetic-ancestry inference on one split at a time using the other nine splits as the reference.

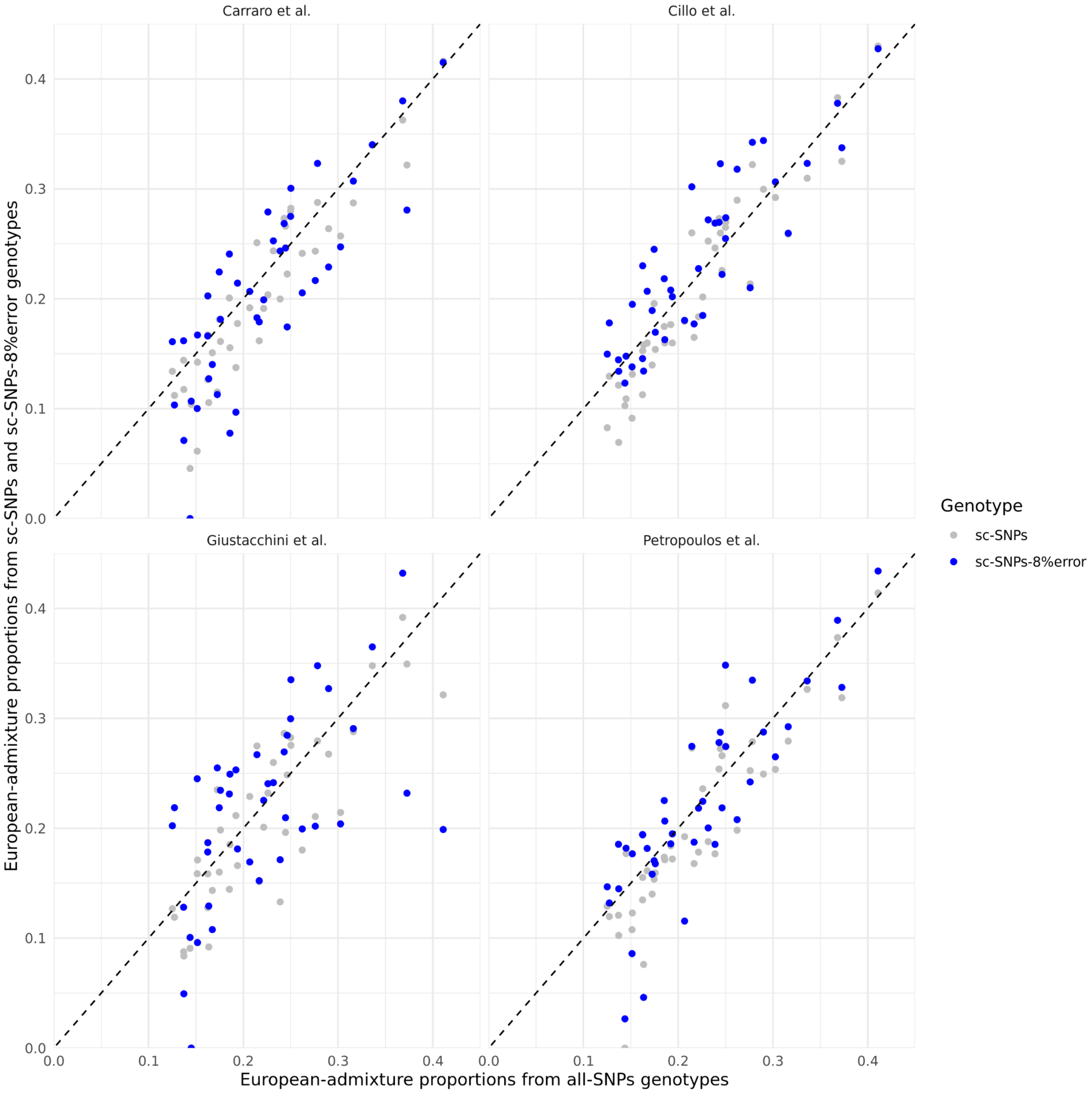

Supplementary Figure 6. ADMIXTURE estimates of European-admixture proportions among ASW individuals stratified by scRNA-seq dataset. We report the results from **Figure 2**, stratified by scRNA-seq dataset.
